# *ARLTS1* polymorphism is associated with an increased risk of familial cancer: evidence from a meta-analysis

**DOI:** 10.1186/s13053-017-0068-7

**Published:** 2017-06-13

**Authors:** Yan Jiang, Chen-Yang Zhao, Li-Chun Cheng, Bing Xu, Hui-Yi Lv

**Affiliations:** Department of Pharmaceuticals, The Second Affiliated Hospital of Dalian Medical University, Dalian, 116027 People’s Republic of China

**Keywords:** *ARLTS1*, Gene polymorphism, Cancer risk, Familial cancer, Meta-analysis

## Abstract

Adenosine diphosphate (ADP)-ribosylation factor-like tumour suppressor gene 1(*ARLTS1*) might be associated with an increased risk of several types of familial cancers. However, previous studies have shown that cancer susceptibility is not completely consistent with *ARLTS1* polymorphisms, and the precise mechanism remains unknown. Therefore, we conducted a meta-analysis of case-control studies by searching the PubMed, Embase, OVID, Science Direct and Chinese National Knowledge Infrastructure (CNKI) databases. In total, 12 studies met the inclusion criteria and were included in this meta-analysis. Statistical analyses were performed using STATA 11.0 software. Overall, the Cys148Arg T > C variant significantly increased cancer risk (CC vs. TT: OR = 1.27, 95% CI = 1.15–1.41, *P* < 0.05). The stratification indicated that the Cys148Arg variant is significantly associated with sporadic cancer (CC vs. TT: OR = 1.36, 95% CI = 1.18–1.55) and familial cancer (CC vs. TT: OR = 1.26, 95% CI = 1.12–1.43). Trp149Stop, Pro131Leu, Ser99Ser and Leu132Leu were not correlated with cancer susceptibility. Based on these results, we demonstrated that the *ARLTS1* Cys148Arg polymorphism is associated with an increased risk of sporadic cancer and familial cancer, and there were no associations between the other four SNPs (i.e., Trp149Stop, Pro131Leu, Ser99Ser and Leu132Leu) and cancer risk.

## Background

Adenosine diphosphate (ADP)-ribosylation factor-like tumour suppressor gene 1 (*ARLTS1*), which is also known as ADP-ribosylation factor-like protein 11 (*ARL11*), islocated in chromosomal region 13q14.3 and is a member of the Alternative Reading Frame (*ARF*) family of the RAS super family of small GTPases, which is involved in apoptotic signalling [1, 2]. *ARLTS1* comprises two exons, its second exon encompasses the entire open reading frame andencodes 196 amino acid guanine-nucleotide-binding proteins that are critical components of several different eukaryotic vesicle trafficking pathways [3].

In 2005, Calin et al. found that chronic lymphocytic leukaemia was associated with a polymorphism in *ARLTS1* [2]. Subsequently, *ARLTS1* variants were shown to be associated with an increased risk of several types of sporadic and familial cancers, such as prostate cancer [4], ovarian cancer [5], breast cancer [6] and other cancers [7]. However, *ARLTS1* variants did not exhibit a significantly increased risk of familial colorectal cancer [8]. Consequently, the results of these studies remain inconsistent. Hence, in the present study, a meta-analysis of all relevant case-control studies published before 1 November 2016 was performed to more precisely estimate the relationship between the *ARLTS1* variants listed in Table 1 and the susceptibility to cancer.

**Table 1:** The different names of the 5 single-nucleotide polymorphisms (SNPs)

Nucleotide variant	Amino acid change
442 T > C	Cys148Arg/C148R/rs3803185
446G > A	Trp149Stop/W149X/rs34301344
392C > T	Pro131Leu/P131L
279G > A	Ser99Ser/S99S/rs3803186
396G > C	Leu132Leu/L132L

## Materials and methods

### Literature search

Searches of the Medline, PubMed, Embase, Web of Science and China National Knowledge Infrastructure (CNKI) databases were performed (from January 2005 to November 2016). The following search words and their combinations were used: (“genetic polymorphism” or “polymorphism” or “SNP” or “gene mutation” or “genetic variants”) and (“cancer” or “melanoma” or “lymphocytic leukaemia” or “carcinoma” or “malignancy”) and “*ARLTS1*”. All identified studies were retrieved, and their bibliographies were reviewed to identify other relevant publications. Review articles were manually searched for additional studies. In the case of overlapping and republished studies, only the most recent study or the study with the largest sample size was selected for this meta-analysis.

### Inclusion and exclusion criteria

The studies included in this meta-analysis met the following criteria: (a) the studies used a case-control design; (b) the studies evaluated the association between *ARLTS1* variants (Cys148Arg, Trp149Stop, Pro131Leu, Ser99Ser and Leu132Leu) and cancer susceptibility; and (c) the studies had sufficient published data to calculate the odds ratio (OR) with a 95% confidence interval (CI). The primary reasons for the exclusion of studies were as follows: (a) no control population was included, (b) the studies contained overlapping data, and (c) the genotype frequency datawere not available.

### Data extraction

Two investigators independently collected the data from all eligible publications according to the inclusion and exclusion criteria. Whenever disagreements occurred, a third investigator was consulted to resolve the dispute, and a final decision was made by the majority of the votes. The following data were extracted from each study: the first author’s last name, publication year, ethnicity of the study population, study design, cancer type, total number of cases and controls and the Hardy-Weinberg equilibrium (HWE) *p*-value. The different cancer types were classified as sporadic tumours or familial tumours. The source of the controls was stratified into hospital-based studies and population-based studies.

### Quality assessment

The quality of the included publications was assessed using a quality assessment scale (Table 2), which was modified from previously published meta-analyses [9–11]. Two investigators assessed the quality of the studies according to the scale for quality assessment, and disagreements were resolved by discussion and consensus. The total scores ranged from 0 to 10. Scores of 0–4,5–8 and 9–10 were defined as low, moderate and high quality, respectively.

**Table 2:** Scale for quality assessment

Parameter	Detail	Score
1.Source of cases	Selected from a population or cancer registry	2
	Selected from an oncology department or cancer institute	1
	No description	0
2.Representativeness of controls	Population	2
	Population-hospital mixed	1.5
	Hospital	1
	No description	0
3.Definition of controls	No history of disease	2
	No description of source	0
4.HWE^a^	Conformity	2
	Inconformity	0
5. Total sample size	>1000	2
	200–1000	1
	<200	0

^a^ If the study did not conform to the HWE (*P* > 0.05), the study was immediately defined as low quality

### Statistical analysis

In our meta-analysis,we investigated the association between *ARLTS1* polymorphisms and cancer risk according to a pooled OR with the corresponding 95% CIs. The stratification analysis was performed by type of cancer, control source and study quality. The significance of the pooled ORs was determined by a Z test, and *P* < 0.05 was considered statistically significant.

In our study, the HWE was tested using the *χ* ^2^ test (significance was set as *P* < 0.05). The heterogeneity of the studies was examined using the Q-test and the I^2^ test [12, 13]. If the between-study heterogeneity was significant (*P* < 0.05 for the Q-test and I^2^ > 50%), we used a random-effects model (DerSimonian-Laird method) [14]; otherwise, the fixed-effects model (Mantel-Haenszel method) was used [15]. The sensitivity was assessed by the omission of individual studies to determine the stability of the results in this meta-analysis. To assess the potential publication bias, a Begg’s funnel plot was generated to visually detect bias [16]. In addition, Egger’s test was also conducted to analyse the publication bias statistically [17]. All *p*-values were two-sided. All analyses were calculated using STATA Version 11.0 software.

## Results

### Characteristics of the included studies

**Fig. 1:**
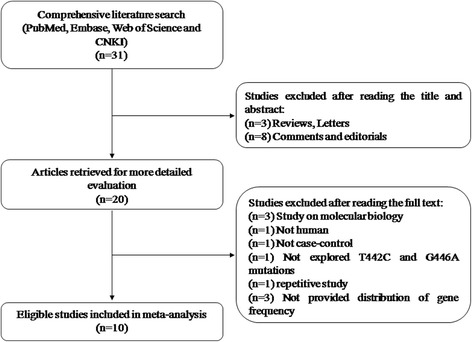
Flow diagram of the study selection process

By searching the databases, 31 abstracts were collected according to the search criteria. Of these 31 studies, 11 studies were excluded after reviewing the titles and abstracts. Another 10 studies were excluded after reviewing the full texts. The study selection process is shown in Fig. 1. Finally, 10 studies met the inclusion criteria and were included in the meta-analysis. Of the 10 studies, one study [18] involved three cohorts, and, thus, each cohort was considered a single study. In total, 12 case-control studies [4, 6, 8, 18–24], evaluating the association between *ARLTS1* polymorphisms and cancer susceptibility, were included in this meta-analysis. The characteristics of all studies are summarized in Table 3. Not all studies genotyped the SNPs included in this meta-analysis. Therefore, the number of included studies differs across the SNPs. All results are presented as the ORs (95% CI), *p*-value.

**Table 3:** Characteristics of the studies included in this meta-analysis

Study	Ethnicity	Type of cancer	Controls source	Case	Control	Variants studied^a^	Study quality
Siltanen [4]	Caucasian	Prostate cancer	Population	2060	760	442 T > C	Low
Akisik [6]	Caucasian	Breast cancer	Population	147	120	446G > A	Moderate
Yang [19]	Asian	Ovarian Cancer	Population	165	120	442 T > C/279G > A	Moderate
Siltanen [18]	Caucasian	Prostate cancer	Population	541	809	442 T > C/446G > A/392C > T	High
Siltanen [18]	Caucasian	Breast cancer	Population	1242	809	442 T > C/446G > A/392C > T	High
Siltanen [18]	Caucasian	Colorectal cancer	Hospital	241	809	442 T > C/446G > A/392C > T	High
Li [20]	Caucasian	Skin carcinoma	Hospital	528	533	442 T > C/446G > A/392C > T/279G > A/396G > C	Moderate
Castellvi-Bel [21]	Caucasian	Colorectal cancer	Hospital	515	515	442 T > C/446G > A/392C > T/396G > C	High
Sellick [22]	Caucasian	Lymphocytic leukaemia	Population	416	471	442 T > C/446G > A/392C > T/279G > A/396G > C	High
Frank [23]	Caucasian	Melanoma risk	Population	351	804	442 T > C/446G > A	High
Frank [24]	Caucasian	Breast cancer	Population	482	530	442 T > C/446G > A/392C > T	High
Frank [8]	Caucasian	Colorectal cancer	Population	611	538	442 T > C/446G > A	High

^a^ Cys148Arg (442 T > C), Trp149Stop (446G > A), Pro131Leu(392C > T), Ser99Ser (279G > A) and Leu132Leu(396G > C)

### Quantitative analysis

#### Cys148Arg

**Fig. 2:**
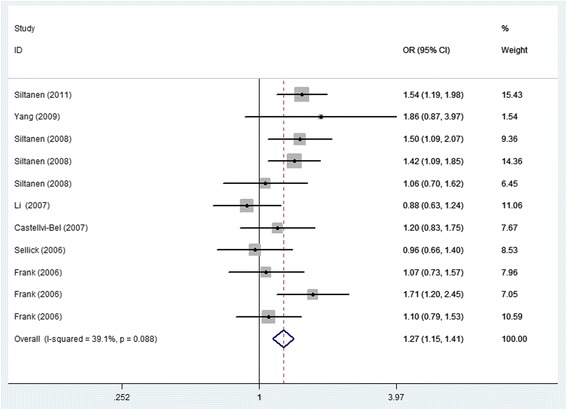
Forest plot of the cancer risk associated with the *ARLTS1* Cys148Arg polymorphism (the genetic model is CC vs. TT)

**Fig. 3:**
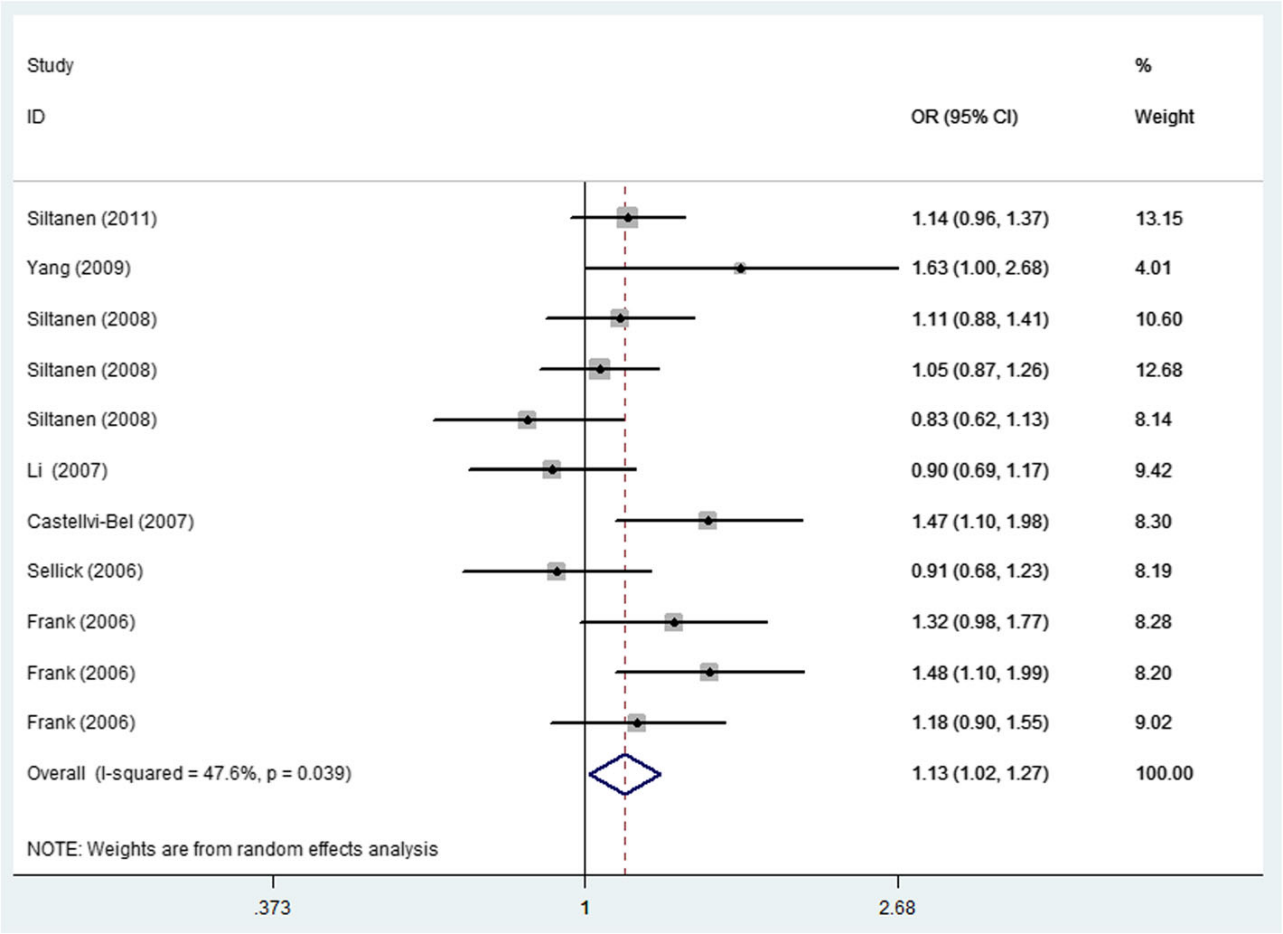
Forest plot of the cancer risk associated with the *ARLTS1* Cys148Arg polymorphism (the genetic model is CC + TC vs. TT)

Among the 12 eligible studies, 11 studies included in this meta-analysis involved SNP C148R with 7152 cases and 6698 controls. Overall, when the 11 studies were pooled into the meta-analysis, the significant main effects on cancer risk were associated with *ARLTS1* Cys148Arg (CC vs. TT: 1.27 (1.15–1.41), *p* = 0.000 Fig. 2; CC + TC vs. TT: 1.13 (1.02–1.27), *p* = 0.026 Fig. 3; CC vs. TC + TT: 1.16 (1.00–1.35), *p* = 0.045). The meta-analysis results are listed in Table 4. Furthermore, several stratified analyses were performed by control source, study quality and type of cancer (Table 4). Regarding the control source, the *ARLTS1* Cys148Arg CC genotype increased the cancer risk in a population-based (CC vs. TT: 1.38 (1.22–1.55), *p* = 0.000; CC + TC vs. TT:1.16(1.03,1.30), *p* = 0.003; CC vs. TC + TT:1.26(1.07–1.49), *p* = 0.000), but not in a hospital-based setting. Regarding the study quality, the results were only significant in the high-quality studies (CC vs. TT: 1.35(1.16–1.57), *p* = 0.001; CC + TC vs. TT: 1.14 (1.00–1.31), *p* = 0.008). In addition, positive results were observed in both familial cancer (CC vs. TT: 1.26 (1.12–1.43), *p* = 0.000; CC + TC vs. TT: 1.15 (1.02–1.29), *p* = 0.021) and sporadic cancer (CC vs. TT: 1.36 (1.18–1.55), *p* = 0.000; CC + TC vs. TT: 1.13 (1.02–1.25), *p* = 0.016; CC vs. TC + TT: 1.22 (1.02–1.46), *p* = 0.030).

**Table 4:** Results of the meta-analysis of the association between the *ARLTS1* Cys148Arg variant and cancer risk

ARLTS1	CC vs. TT		TC vs. TT		CC + TC vs. TT		CC vs. TC + TT	
Cys148Arg	OR (95% CI)	P_h_	OR (95% CI)	P_h_	OR (95% CI)	P_h_	OR (95% CI)	P_h_
Total	1.27(1.15,1.41)	0.088	1.10(0.96,1.25)	0.008	1.13(1.02,1.27)	0.039	1.16(1.00,1.35)	0.003
Controls source								
Population	1.38(1.22,1.55)	0.330	1.10(0.95,1.27)	0.033	1.16(1.03,1.30)	0.135	1.26(1.07,1.49)	0.019
Hospital	1.00(0.81,1.23)	0.476	1.09(0.76,1.57)	0.012	1.06(0.78,1.45)	0.027	0.94(0.79,1.12)	0.739
Study quality								
High	1.35(1.16,1.57)	0.264	1.10(0.94,1.32)	0.005	1.14(1.00,1.31)	0.049	1.14(0.96,1.35)	0.009
Low & Moderate	1.30(1.07,1.58)	0.023	1.05(0.84,1.31)	0.185	1.12(0.87,1.45)	0.088	1.24(0.86,1.79)	0.037
Type of cancer								
Familial	1.26(1.12,1.43)	0.060	1.11(0.93,1.33)	0.050	1.15(1.02,1.29)	0.065	1.21(0.98,1.49)	0.018
Sporadic	1.36(1.18,1.55)	0.797	1.08(0.91,1.29)	0.022	1.13(1.02,1.25)	0.203	1.22(1.02,1.46)	0.050

*OR* odds ratios, *95% CI* 95% confidence interval, P_h_ *p*-values for heterogeneity in Q-test

#### Trp149Stop, Pro131Leu, Ser99Ser and Leu132Leu

All studies were pooled in the meta-analysis. No significant results were observed for any of these SNPs (Trp149Stop GA vs. AA: 1.11 (0.66–1.86), *p* = 0.700; Pro131Leu CT vs. CC: 0.94 (0.81–1.08), *p* = 0.374; Ser99Ser AG vs. AA: 1.02 (0.86–1.20) *p* = 0.847; Leu132Leu GC vs. GG: 0.90 (0.58–1.39) *p* = 0.845), which are referred to in Table 5. Furthermore, the stratified analysis by familial and sporadic cancer revealed no association between these variants and cancer.

**Table 5:** Results of the meta-analysis of the association between the *ARLTS1* Trp149Stop, Pro131Leu, Ser99Ser and Leu132Leu variants and cancer risk

ARLTS1	Total	Familial	Sporadic
polymorphism	OR (95% CI) P_h_	OR (95% CI) P_h_	OR (95% CI) P_h_
Trp149Stop			
GA vs. AA	1.11(0.66,1.86) 0.001	1.14(0.76,1.72) 0.843	0.65(0.38,1.10) 0.376
GG + GA vs. AA	1.13(0.68,1.88) 0.001	1.20(0.80,1.79) 0.852	0.65(0.38,1.10) 0.376
Pro131Leu			
CT vs. CC	0.94(0.81,1.08) 0.624	1.14(0.76,1.72) 0.843	0.65(0.38,1.10) 0.376
TT + CT vs. CC	0.93(0.81,1.07) 0.559	1.20(0.80,1.79) 0.852	0.65(0.38,1.10) 0.376
Ser99Ser			
AGvs. AA	1.04(0.73,1.48) 0.340	1.72(0.88,3.37) 0.943	1.35(0.40,1.79) 0.031
GG + AGvs. AA	1.05(0.75,1.47) 0.366	1.67(0.93,3.02) 0.898	0.84(0.44,1.64) 0.043
Leu132Leu			
GC vs. GG	1.10(0.42,2.68) 0.674	1.13(0.26,4.90) 0.150	1.55(0.47,5.13) 0.000

*OR* odds ratios, *95%CI*, 95% confidence interval, *P*_h_, *p*-values for heterogeneity in Q-test

#### Sensitivity analysis and publication bias

**Fig. 4:**
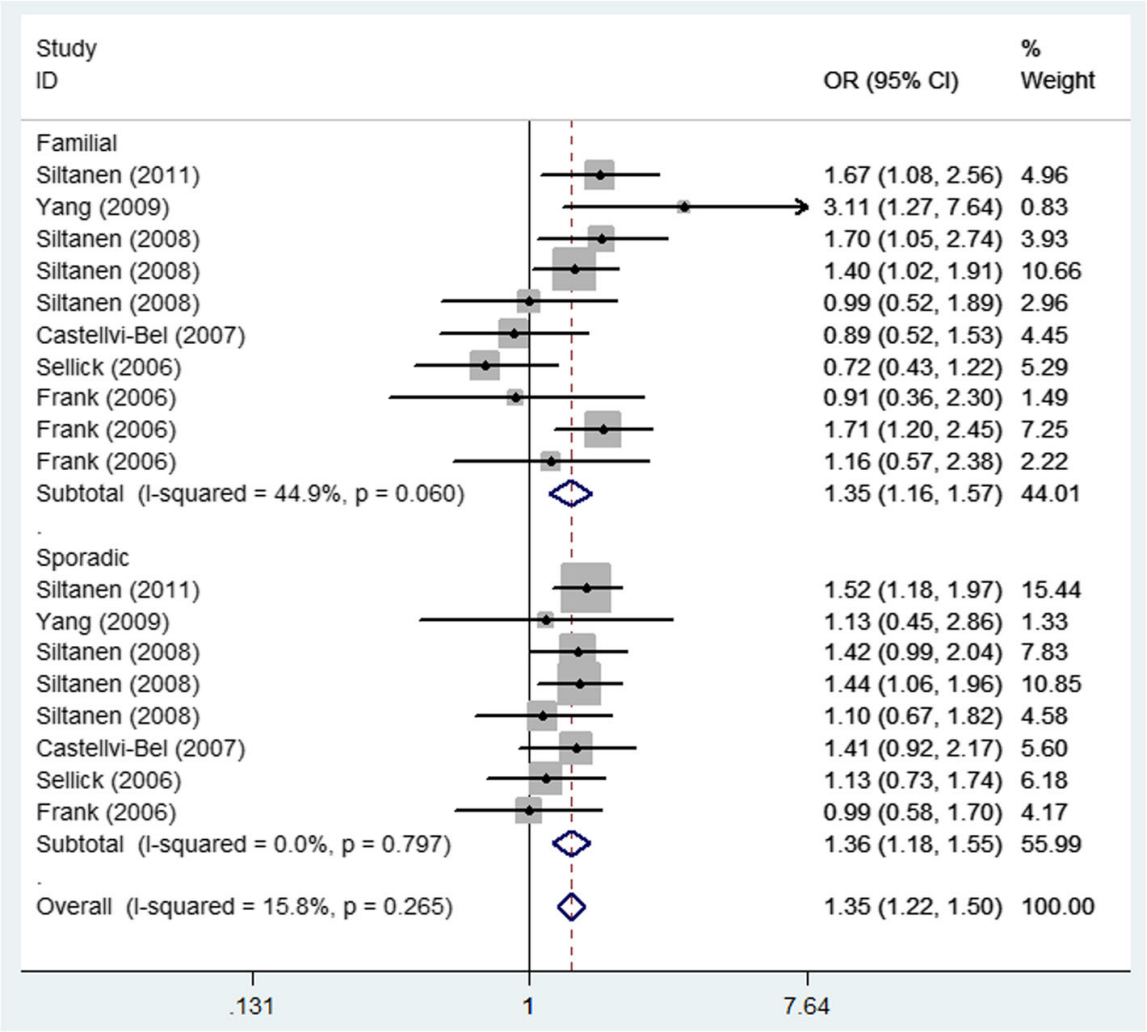
Forest plot of the familial and sporadic cancer risk associated with the *ARLTS1* Cys148Arg polymorphism (the genetic model is CC vs. TT)

**Fig. 5:**
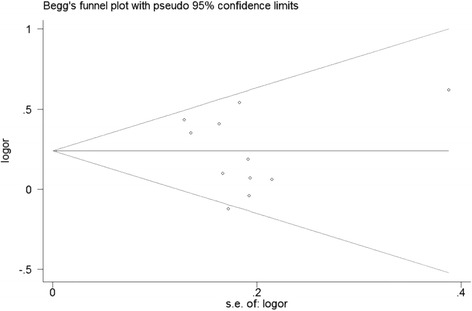
Funnel plot of the association between the *ARLTS1* Cys148Arg polymorphism and cancer risk (the genetic model is CC vs. TT)

Each study was evaluated in this meta-analysis individually to detect the influence of the individual data set on the pooled ORs. The results were not substantially altered, indicating that our results were stable and robust. Begg’s funnel plots were generated to assess the publication bias, and the shapes revealed no evidence of obvious asymmetry (Fig. 4). Egger’s test was performed based on the linear regression of the standard normal deviation against its precision, which was performed to test for the existence of publication bias. The results did not show any statistical evidence of publication bias for *ARLTS1* Cys148Arg (*p* = 0.595 for CC vs. TT) (Fig. 5).

## Discussion

*ARLTS1* is a cancer-associated gene with notable tumour suppressor properties. *ARLTS1* has been shown to induce apoptosis [25]. However, the precise functional properties of the *ARLTS1* gene remain unclear, such as its access, interactions in the 13q14 region and the structure of the protein. The proposed mechanism is that the *ARLTS1* gene produces proteins that affect acceptors of the immune system via transcription and translation, and then, endogenous inflammatory cells become activated to suppress tumour formation. The *ARLTS1* gene may have a restraining effect on cancer development in lung [26] and ovarian cancer [5]. The expression of *ARLTS1* is frequently down-regulated in prostate cancer [25] and chronic lymphocytic leukaemia samples [27]. The potential mechanisms leading to the down-regulated expression of *ARLTS1* include promoter hypermethylation or loss of heterozygosity (LOH) in the gene region. The reduction or absence of *ARLTS1* expression has been reported to contribute to DNA mutations with an LOH in breast cancer and a mutation with methylation in thyroid cancer [2]. This down-regulated expression immediately impacts immune responses, resulting in diminished apoptosis, reduced defence mechanisms and cancer progression. Additionally, the *ARLTS1* polymorphism was not only associated with sporadic cancer but also with familial cancer [28]. Consequently, the association between *ARLTS1* polymorphisms and cancer risk, particularly familial cancer, has recently become a high priority research topic.

To the best of our knowledge, the present study is the first systematic review to investigate the relationship between the *ARLTS1* polymorphisms and cancer risk based on 12 case-control informative studies. Regarding Cys148Arg, we found that the SNP C148R polymorphism was significantly associated with cancer in all subjects. When a subgroup analysis was carried out based on the size of the total sample, we observed different associations with sporadic cancer and familial cancer, which was consistent with the result obtained by Calin et al [2]. Regarding Trp149Stop, Pro131Leu, Ser99Ser and Leu132Leu, however, we found no correlations with cancer risk. Therefore, we speculate that the *ARLTS1* Cys148Arg CC genotype may significantly contribute to decreased apoptosis, thereby promoting the overall cancer incidence. However, the other variants may still have associations with cancer. These variants may have minor effects individually, but they are likely to contribute adequately in combination to lead to the failure of theimmune response. Therefore, gene-gene interactions should be considered in future studies to elucidate the precise role of *ARLTS1*.

Some limitations of this meta-analysis should be addressed. First, this study did not analyse the potential and gene-environment interactions due to a lack of original data, such as data involving environmental risk factors and genotypes. Second, the controls were not uniformly defined. Although most of the controls were selected mainly from healthy populations, some controls had a benign disease. In addition, our results were based on unadjusted estimates without adjusting for other risk factors, such as age, smoking status, drinking status, obesity and environmental factors. Therefore, a more precise analysis should be conducted if individual data were available. Although this study has several limitations, as previously stated, this study is the first to reveal the association between Cys148Arg and cancer risk and exclude the association between the other four SNPs (i.e., Trp149Stop, Pro131Leu, Ser99Ser and Leu132Leu) and cancer risk. Additionally, our study provides powerful evidence for future large-scale population-based cohort and case-control studies.

## Conclusion

In summary, the *ARLTS1* Cys148Arg polymorphism was associated with sporadic cancer risk and familial cancer risk. However, well-designed studies with larger sample sizes should be performed to confirm this association. Moreover, further studies estimating the effect of gene-gene and gene-environment interactions may eventually provide a better and more comprehensive understanding to clarify the role of *ARLTS1* in the pathogenesis of cancer.
